# POSTOPERATIVE EFFECT OF PHYSICAL THERAPY RELATED TO FUNCTIONAL CAPACITY AND RESPIRATORY MUSCLE STRENGTH IN PATIENTS SUBMITTED TO BARIATRIC SURGERY

**DOI:** 10.1590/0102-6720201600S10012

**Published:** 2016

**Authors:** Josélia Jucirema Jarschel de OLIVEIRA, Alexandre Coutinho Teixeira de FREITAS, Andréa Adriana de ALMEIDA

**Affiliations:** Postgraduate Program in Surgical Clinics, Health Science Department, Clinic Hospital, Federal University of Paraná, Curitiba, PR, Brazil.

**Keywords:** Obesity, Physical Therapy, Exercise, Bariatric Surgery

## Abstract

**Background:**

Respiratory physiotherapy plays an important role preventing complications in bariatric surgery.

**Aim:**

To assess the effects of out-patient physiotherapy during post-operative period through respiratory pressures and functional capacity in individuals submitted to bariatric surgery.

**Method:**

A prospective longitudinal and controlled study was done in adults with body mass index (BMI) equal or greater than 40 kg/m², who have been submitted to bariatric surgery. They were divided into two groups: intervention-group, who performed out-patient physiotherapy twice a week, from thirty to sixty days after surgery; and the control-group, who only followed home instructions. Both groups were evaluated before surgery and sixty days after surgery through manovacuometry, six-minute walk test and the Borg Scale of perceived exertion.

**Results:**

Twenty participants were included the intervention-group and twenty-three in the control-group. Both groups had significant and similar weight loss after surgery. The manovacuometry presented no differences comparing pre- and post-surgery and in the comparison between the groups. The result of the six-minute walk test for the intervention-group increased by 10.1% in the post-operative period in relation to pre-. The Borg scale of perceived exertion in the intervention-group in pre-surgery decreased by 13.5% in the post-surgery compared to pre-surgery. In the control-group there was no difference comparing pre- and post-operative values, as in the comparison with the intervention-group.

**Conclusion:**

The low-intensity exercise program, carried out between the 30^th^ and the 60^th^ day after bariatric surgery provided better functional capacity; did not change respiratory muscle strength; and improved the perceived exertion rate.

## RESUMO

**Racional:**

A fisioterapia respiratória tem papel importante na prevenção das complicações da cirurgia bariátrica.

**Objetivo:**

Avaliar os efeitos da fisioterapia ambulatorial no pós-operatório através das pressões respiratórias e da capacidade funcional dos indivíduos submetidos à cirurgia bariátrica.

**Método:**

Estudo prospectivo, longitudinal, randomizado e controlado, em adultos com IMC≥40 kg/m², que se submeteram a cirurgia bariátrica, Foram divididos em dois grupos: grupo intervenção, que realizou fisioterapia ambulatorialmente, duas vezes por semana, do trigésimo ao sexagésimo dia de pós-operatório; e grupo controle, que seguiu orientações domiciliares. Ambos os grupos foram avaliados no pré-operatório e após 60 dias da operação através das pressões respiratórias com a manovacuometria, do teste da caminhada de 6 min e do índice de percepção de esforços pela escala de Borg.

**Resultados:**

Foram incluídos 20 participantes no grupo intervenção e 23 no grupo controle, ambos com perda de peso significativa e similar no pós-operatório. A manovacuometria não demonstrou diferenças nas pressões respiratórias na comparação entre o pré-operatório e o pós-operatório e na comparação entre os grupos. O resultado do teste da caminhada de 6 min para o grupo intervenção aumentou em 10,1% no pós-operatório em relação ao pré-operatório. A percepção de esforço pela escala de Borg no grupo intervenção reduziu em 13,5% no pós-operatório comparado ao pré. No grupo controle não foi observada diferença entre o pré e o pós-operatório bem como na comparação com o grupo intervenção.

**Conclusão:**

O programa de exercícios de baixa intensidade realizado entre o 30º e o 60º dia de pós-operatório de cirurgia bariátrica promoveu melhor capacidade funcional; não modificou a força muscular respiratória; e melhorou o índice de percepção de esforço.

## INTRODUCTION

Obesity may be defined by excessive accumulation of body fat. To the date, it is considered as a risk factor upon several conditions. It is a matter of public health, as genetically and metabolically descendant, aggravated by the exposure to environmental, cultural, social and economic conditions, once related to demographic factors (gender, color, age) and sedentary behavior^13^ ^,^ ^18^.

Generally speaking, obesity classification is rendered by the body mass index (BMI) as calculated by dividing the weight in kilograms towards the square of height in meters. In 1995 the World Health Organization^22^ embraced the BMI ratio as a measure set for obesity and its classification arranged as class I for the BMI between 30-34.9 kg/m^2^; class II from 35-39.9 kg/m^2^ and class III or morbid obesity for the BMI parameters equal or higher than 40 kg/m^2^. The above mentioned institution^23^ estimates a spread of 300 million obese worldwide. In Brazil it is estimated a range of 606,000 people inserted in morbid obesity, with prevalence in females.

The usual treatment applied to obesity is a low-calorie diet, physical activity, psychological assistance and medications. Bariatric surgery is indicated for the ones with the BMI equal or higher than 40 kg/m² or BMI from 35-39.9 kg/m² in association with chronic diseases once triggered or aggravated by obesity. Other factors may also be highlighted, as to the potential ones from ages between 18-65, who may furnish evidences in failure of conservative treatments regularly performed for at least a two years, and showing motivation and acceptance as well as awareness on the given surgical risks. The candidates for the procedure may not carry on contraindications, such as: endocrine treatable root causes for obesity; alcohol or illicit drugs addiction; severe non-prescribed psychiatric condition; ASA-IV anesthetic and surgical risks incurred and uneasiness to cope with the risks, benefits, expected results, alternative treatments and changes in lifestyle if triggered by bariatric surgery^1^.

In perioperative timeframe, the physiotherapist, part of the multidisciplinary team, is entitled to identify the risk factors, taken through history and physical examination, and mainly to prevent pulmonary and circulatory issues. 

The purpose of this essay was to analyze the effects of a postoperative low-intensity activity program carried out between the 30^th^ through the 60^th^ day in regard to functional capacity, respiratory muscle strength and perceived exertion index. 

## METHOD

The study is prospective, longitudinal and randomized in which obese adults who underwent to bariatric surgery were evaluated in preoperative and postoperative, assorted into two similar groups, one with physical therapy intervention up to the 30^th^ day until the completion of the 60 days, and the other under non-interventional measure, solely being observed in the same postoperative period. The research project was approved by the Ethics Committee on Research in Human Beings from HC-UFPR and registered in Brazil Platform upon CAAE n^o^. 021297120.0000.0096 and upon Protocol n^o^. 24457/2012.

The patients were enrolled from the Obesity Clinic of the Clinic Hospital of Federal University of Paraná, Out-patient Medical Service from July 2012 to March 2014. 

The inclusion criteria were: adults, from ages between 18-60, both genders, class III obesity ranked, BMI ≥40 kg/m^2^, enrolled in the waiting queue for laparoscopic Roux-en-Y gastric bypass^19^, eligible to remain without limiting factors and stay in the city in the postoperative period. The exclusion criteria were: severe heart condition, severe infection, limiting arthropathies and medical contraindication in performing physical activities.

All participants were evaluated in the preoperative phase, and revaluated after 60 days carried on from the postoperative phase by means of a six-minute walk test (6MWD)^2^, as per the ratio of maximum inspiratory and expiratory pressure taken by manovacuometry and by the Borg scale of perceived exertion (EPSEB)^17^.

Manovacuometry was held in sitting-position with Wika(r) analogic manovacuometer type, ranging between -120/+120 cm H_2_O in a scale of 4 cm H_2_O, with Rescal silicone adapter for manual maneuver, with a mouth piece with an orifice at the distal end which is sealed during inhaling, facilitating the exact time to be measured^7^. The test was explained in a simple and objective manner, for the proper comprehension of the patient to exhale all air and then to inhale with occluded mouth with the mouthpiece between the teeth, and occluded hole. As per the maximum inspiratory pressure (MIP), in the same maneuver, it was measured the maximum expiratory pressure (MEP) through forceful exhalation. Such maneuver as performed five times each patient, with 10 seconds of interval, and then taking notes on MIP and MEP higher flow rates. For each analysis it was applied a sterile connector^7^. For the sake of proper comprehension it was calculated the pressure rates in both MIP and MEP, as run by Neder´s regression equations (Table 1). 

**TABLE 1:**
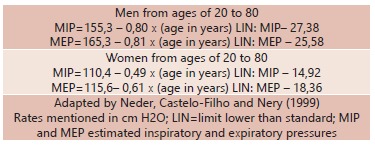
Regression equation for calculating MIP and MEP of the Brazilian population, according to age and gender

The 6-minute walk test was held in accordance with established directives from American Thoracic Society (ATS) upon the patients´ acquiescence^2^ ^,^ ^8^. In a place as previously scheduled for the test to be held, the supervisor explained the purpose and the route to be performed, advising that the test could be interrupted at any point time if necessary.

Preliminary beginning the agent inquired the patient in order to describe the fatigue level as Borg scale of perceived exertion (EPSEB). The 6MWD was applied in an in door corridor, with little traffic of people, with proper window ventilation in its entire length, with hard rectilinear floor padding. A 30 m length strip line tape was marked on the floor. In every 3 m there was a marking according to the recommendations of the American Thoracic Society. Before and after 6MWD the patient was monitored by cardio, by respiratory frequency, by peripheral oxygen saturation, by blood pressure and EPSEB^2^ ^,^ ^5^ ^,^ ^7^ ^,^ ^8^.

The walk test lasted for 6 m, tracked by chronometer. It was recorded in a separate form the number of complete and incomplete laps along the track. For each completed lap the professional agent encouraged the patient as "very good", "keep on going". It was applied for the predicted values ​​and the calculation of 6MWD, the reference equation providing an estimation of the distance in the six-minute walk test, as Enright and Sherril (1998). For male patients the estimated distance was equal to (7.57 cm x height) - (5.02 x age) - (1.76 x weight kg) - 309 m, minus 153 m in order to acquire the lower standard limit. For female the ratio was (2.11 x height cm) - (2.29 x weight kg) - (5.78 x age) + 667 m, minus 153 m for the lower standard limit.

Afterwards, it was applied the Borg scale showing it in laminated paper with numerical values from ​​6 to 20 in ascending order in correspondence from grey to red. This number represented the heartbeat of 60-200 bpm, which defines the effort shown by each participant.

In the postoperative phase, the hospital physical therapy was carried out for all patients and with certain emphasis on respiratory, circulatory functions, around three days when discharged from the hospital, and randomization of the sample held by a raffle.

Participants in the intervention group resumed therapy sessions on the 30^th^ day after surgery after medical release with use of abdominal elastic strap.

In physical therapy clinic there was the development of a customized program. Participants performed a 10 min walk as awarm-up, taking into consideration each individual rhythm. Then, it was performed breathing exercises with diaphragmatic breathing with deep inhales and exhales with pursed lips, and three-phased fractioned inhalation in order to improve thoracic expansion simultaneously to the flexion and extension of the upper limbs. For the sake of exercise optimizer it was used wooden sticks with 1 m long and without significant weight. Also for the movement of adduction and abduction of the shoulder it was used 1 kg of weight as held by the hands of the patient. The legs also followed the pattern of flexion, extension, adduction and abduction free from weight. These ones, however, respected the hip joint, not compressing the abdomen, and it was held in the lying and standing position. Then, in the sitting position, it was performed arms stretching along the body, diaphragmatic breaths, and trunk bending forward in order to relax and complete the exercises. The duration of the sessions took around 40 min, with two sessions per week and always 15 repetition on each activity^10^ ^,^ ^14^. For financial and economic issues none of the surveyed participants had use of apparel breathing exercises. After 60 days of postoperative elapsed, the participants were re-evaluated under the same tests.

The control group (guided) after release they were instructed to perform physical activities in the postoperative timetable basis, such as not lying all daylong, to walk for half an hour three times a week, performing flexion-extension ankles whenever is seated or bended in resting position for at least 20 times. Such group was also evaluated after reaching 60 days of postoperative condition, through the same tests from the intervention group.

Once data was collected in both groups the maximum respiratory pressures, 6MWD and EPSEB were taken into comparison

## RESULTS

There were 103 patients evaluated as prone to perform bariatric surgery and 43 were inserted in the study. Sixty individuals were excluded, 48 did not take the procedure, two had postoperative complications, 10 did not agree to participate.

From the 43 participants, 95.34% were female and 4.65% male, age in average of 37.27±12.04. All of them were ranked under class III obesity. As per risk factors 37.2% were smokers with medical advice to quit smoking in at least one month before the procedure; hypertensive in treatment in 46.51%; diabetes mellitus type II in 27.9%; arthralgia in 58.13%; and dyspnea on exertion in 18.6% (Table 2). Twenty patients were randomized for the intervention group and 23 for the control group. Out of the 43 mentioned, six of them did not incurred on the second test.

**TABLE 2:** Anthropometric sample data

Sample features(n=43)	Percentage
Age in average 37.27±12.04	-
Female	95.34
Male Smokers	4.65 37.2
Hypertensive	46.51
Diabetes type II	27.9
Arthralgia	58.13
Dyspnea on exertion	18.6

The initial weight average from the intervention group was 113.6±12.3 kg and after 60 days elapsed from postoperative the range of 95.6±11.3 kg (p=0.0001). As per the control group the initial weight average was 122.4±19.6 kg and the final 100.1±17 kg (p=0.0005). There was significant weight loss in both groups. 

In regard to BMI, in intervention group the initial average taken was 44,9±4,7 kg/m² and the final average on around 37.8±4.4 kg/m² (p=0.0001); in control group the initial average reached 46.4±5.4 kg/m² and in final average on around 38.3 5.4 kg/m² (p=0.0001).

In intervention group the initial MIP was an average of -84.7±16.2 cm H_2_O and final average of -96.5±19 cm H_2_O (p=0.0661, Table 3). In control group it was noticed in the initial MIP an average of -94.5±22.8 cm H_2_O and final average of -105±18.2 cm H_2_O (p=0.1271). When the groups were put in comparison there was no difference highlighted as per initial and final treatment.

**TABLE 3:** Comparison in MIP intervention groups

Variables/Groups	n	Average (standard. deviation)	p
MIP (cm H2O)			
Initial intervention group	20	-84.7 ± 16.2	0.0661
Final intervention group	17	-96.5 ± 19	

U Mann-Whitney Test

When the maximum inspiratory pressure was calculated per Neder´s equation (1999) (Table 1) it was gathered an estimated MIP of -78.3±6,6 cm H_2_O; both groups presented higher rates than expected. 

In comparison the expiratory pressures it was obtained similar results. In MEP intervention group, the initial average was around 78.2± 18.8 cm H_2_O, and the final average on around 86.5±17.7 cm H_2_O (p=0.3141). In the control group the initial average was 77±52.4 cm H_2_O and the final average was 86±20.6 (p=0.9888). Between both groups there was no difference (Table 4). 

**TABLE 4:** Comparison between MEP groups (cm H2O)

Variables/Groups	n	Average (standard. deviation)	p
Initial MEP (cm H2O)			
Control Group	23	77±52.4	0.2404
Intervention Group	20	78.2±18.8	
Final MEP (cm H2O)			
Control Group	20	86±20.6	0.8923
Intervention Group	17	86.5±17.7	

U Mann-Whitney Test

Regarding MEP estimated flow rates, it was also calculated via Neder´s (1999) (Table 1) and it was obtained a result of 76.2±10.01, rates near to the appraised ones. 

In regard to the six-minute walk test, it was noticed that previously to the procedure the control group walked an average of 424.5±92 m and the intervention group peaked around 408,1±50,3 m. There was no significant difference between walked figures (p=0.6866). The same structure was noticed after the procedure where the control group took an average of 422.7± 56.3 m and the intervention group 449.4± 43.6 m, p=0.0718. 

Yet, it was found the intervention group had initially walked and average of 408.6±48 m, and in the final an average of 449.4± 43.6 (p=0.0151), with a significant difference in terms of statistics. As per the control group there was no difference from the initial 6MWD around 424.5±96.8 m and the final with 422.4±56.3 (p=0.7452, Table 5). 

**TABLE 5:** Comparison of initial and final rates from the same group

Variables/Groups	n	Average (standard. deviation)	p
6MWD (m)			
Initial Control Group	20	424.5±96.8	0.7452
Final Control Group	20	422.7±56.3	
6MWD (m)			
Initial Control Group	17	408.6±48	0.0151
Final Control Group	17	449.4±43.6	

U Mann-Whitney Test

It was rendered Enright and Sherril´s (1998) calculation distance expected equation to all participants, being in preoperative average of 503.77± 85.9 m and in postoperative 568±22.20 m; in both phases the actual results was lower than expected^9^.

In regard to Borg scale of perceived exertion, in the intervention group the perception from initial exertion was around 14.1±1.5 and in the final it was reduced to 12.2±1 (p=0.0007). In the control group the initial perceived exertion was around 13.2±1.7 and in the final one was around 12.2±1.5 (p=0.0623, Table 6). In the comparison between the assessed groups the initial perceived exertion was not different from the control group (p=0.1208), the same pattern took place with final perceived exertion (p=0.7857).

**TABLE 6:** Comparison of perceived exertion rate (Borg) initial and final rates from the same group

Variables/Groups	n	Average (standard. deviation)	p
Borg			
Initial Control Group	20	13.2±1.7	0.0623
Final Control Group	20	12.2±1.5	
Borg			
Initial Control Group	17	14.1±1.6	0.0007
Final Control Group	17	12.2±1	

U Mann-Whitney Test

## DISCUSSION

The purpose of this essay was to verify the respiratory performance, as well as physical features from the participants after 60 days from bariatric surgery, with physical therapy monitoring, in a sample presenting multiple risk factors incurred by obesity.

From the scope of Segal and Fandiño^19^, obesity is considered as a chronic condition caused by multiple factors and its treatment requires various types of approaches. Dietary guidance, physical activity and the use of anti-obesity drugs are the key features for the treatment. However, conventional treatments may point as unsatisfactory for Class III obesity; it is observed that the initial weight is regained in 95% within two years. Due to the necessity for a more effective intervention in the cases of morbid obesity, an indication of bariatric procedure has been growing in to the date. The participants from this study have taken part of such obesity context as well as on postoperative recovery.

In revision Carpio *et al.* ^6^ had described respiratory disorders; such study, held by electromyography, had shown an increase in diaphragmatic function, however it was not evidenced any increase in inspiratory pressure, being noted certain ineffectiveness of muscle contraction. A secondary muscle hypertrophy was evidenced, once assuming the overweight condition to require an overload to the body in reply to the increased breathing function. In the present essay it was established the respiratory parameters from participants who underwent to bariatric surgery from preoperative phase up to 60 days after surgery. Bearing in mind the context of respiratory disorders which follows such obese, it was then agreed to overview the great weight loss phase, and its physical rehabilitation, and to share similar point of views to evaluated respiratory forcefulness and there was not changes in pressure.

Another study as described by Barbalho-Moulim *et al.* ^4^ run with 24 obese participants with similar anthropometric features similar to this study,; it was analyzed the preoperative as well as the postoperative with spirometry and maximum respiratory pressures, obtained negative results which had shown a decrease of the flow rates ​​on both pressures; however, it was not prescribed any postoperative physical therapy, it is believed that this is due to loss of lean muscle mass along with the fat mass. These results are similar to the study under discussion where the results were maintained, only showing the variation of MIP.

Forti *et al* ^12^ reviewing obese and eutrophic participants after inserted in three different physiotherapy methods, concluded that MPI rates were higher in the group of obese. In the meantime MEP rates were similar in both groups. Such report corroborates in certain aspects upon the present study, where MIP rates appeared in a higher status, as opposed to MEP rates which remained than expected. 

In regard to functional capacity, it was rendered the six-minute walk test (6MWD) as well as the EPSEB, which was also mentioned in other studies. Troosters, Gosselnk and Decramer^20^ had applied the 6MWD in different samples, without respiratory problems as indicated by ATS^2^. In the current literature, there are no specific tests prescribed for obese people; however, in some studies the 6MWD was rendered to be performed with obese due to its easiness to apply. Valuable to note, the predictive indexes cannot be considered, as they are applied for standard individual without body changes.

Marcon, Gus and Neumann^16^ had developed a study on monitoring the obese participants in pre- and postoperative throughout six months with low intensity physical activity, provided by walking tests and stretching, as it could be observed an evolution of low exercise tolerance from the preoperative to a significant improvement in functional capacity on postoperative phase. This study, once researching the use of the 6MWD as an evaluation index, had shown an average increase of 12.83% from the previous distance, evidencing an improvement over functional capacity of these participants. As per this study, the travelled distance from pre and post-training was increased by 10.12% from the initial figure (p=0.0151), which corroborates with the study above described, showing an improving pattern over the travelled distance.

Ortega, Juan e Garcia^17^ had sourced an exercise program for post-surgical obese patients, and functional capacity test 6MWD was taken, and similar rates were found as this study. With aerobic activities performed in 16 sessions, it was managed to increase the distance in 6.3% higher than the initial one, demonstrating an improvement on the functional capacity. In the given study, it was set a program similar to the one described, but with a few number of sessions; yet, it was found a pattern in view of the increased distance.

In revision, Fonseca-Junior *^_et al.11_^* highlighted that in wide range of articles there is not agreement upon the method taken to define the platform of physical activities prescribed for obese patients; it is only known the relevance of it, as well as in its positive effects provided on postoperative physical activity prescription-based under obese patients. Therefore, pursuant the authors of this essay, due to the lack of program referral to lean forward to the prescription of appropriate physical activities for obese patients, it was chosen to acustomize low-intensity activity program, not focusing on weight loss but to respiratory integrity and restoration of functional capacity^3^ ^,^ ^11^ ^,^ ^14^ *.*

Faintuch *et al.* ^10^ had concluded physical therapy to be recommended in the postoperative phase in Brazil, as opposed to other countries, since the main focus is the nutritional and metabolic status. Healthy eating and physical activity have been recommended for weight maintenance after bariatric surgery for up to 36 months. This highlights this study saying that postoperative monitoring tend to bolster better household and professional lifestyle. 

## CONCLUSION

The low-intensity activity program, carried out between the 30^th^ and the 60^th^ day after bariatric surgery provided better functional capacity; it did not change the respiratory muscle strength; and it improved the perceived exertion rate. 
